# A vault ribonucleoprotein particle exhibiting 39-fold dihedral symmetry. Corrigendum

**DOI:** 10.1107/S0907444909005824

**Published:** 2009-02-20

**Authors:** Koji Kato, Hideaki Tanaka, Tomoyuki Sumizawa, Masato Yoshimura, Eiki Yamashita, Kenji Iwasaki, Tomitake Tsukihara

**Affiliations:** a Insutitute for Protein Research, Osaka University, 3-2 Yamada-oka, Suita 565-0871, Japan; b Department of Environmental Toxicology, Institute of Industrial Ecological Sciences, University of Occupational and Environmental Health, 1-1 Iseigaoka, Yahatanishi, Kitakyushu 807-8555, Japan; c Bio-multisome Research Team, Structural Physiology Research Group, RIKEN Harima Institute, Mikazuki Sayo, Hyogo 679-5148, Japan; d Core Research for Evolution Science and Technology, Japan Science and Technology Agency, Japan

**Keywords:** vault, ribonucleoproteins, corrigendum

## Abstract

Corrections are made to the article by Kato *et al.* [*Acta Cryst.* (2008). D**64**, 525–531].

## Abstract 2

A corrigendum to the article by Kato *et al.* [*Acta Cryst.* (2008). D**64**, 525–531].

In the article by Kato *et al.* (2008 ▶), the values for ϕ and ψ were transposed. Throughout the paper (ϕ, ψ) = (90°, 110°) should be replaced by (ϕ, ψ) = (110°, 90°). Also, in Fig. 4 of the article the label ϕ in parts (*a*) and (*b*) should be replaced by ψ.

In addition, on page 530 of the article the size of the vault particles was given as 400 × 40 × 700 Å when it should be 400 × 400 × 700 Å.
